# Prevalence and maternal determinants of early and late introduction of complementary foods: results from the Growing Up in New Zealand cohort study

**DOI:** 10.1017/S000711452200112X

**Published:** 2023-02-14

**Authors:** Sara Silva Ferreira, Dirce Maria Lobo Marchioni, Clare Rosemary Wall, Sarah Gerritsen, Juliana Araujo Teixeira, Cameron C. Grant, Susan M. B. Morton, Teresa Gontijo de Castro

**Affiliations:** 1 Department of Nutrition, School of Public Health, University of Sao Paulo, Brazil; 2 Nutrition Section, Faculty of Medical Sciences, University of Auckland, New Zealand; 3 Department of Epidemiology and Biostatistics, School of Population Health, University of Auckland, New Zealand; 4 Centre for Longitudinal Research, School of Population Health, University of Auckland, New Zealand; 5 Department of Paediatrics: Child and Youth Health, School of Medicine, University of Auckland, New Zealand; 6 General Paediatrics, Starship Children’s Hospital, Auckland, New Zealand

**Keywords:** Timing of food introduction, Infant feeding, Complementary foods, Nutritional Epidemiology

## Abstract

A nationally generalisable cohort (*n* 5770) was used to determine the prevalence of non-timely (early/late) introduction of complementary food and core food groups and associations with maternal sociodemographic and health behaviours in New Zealand (NZ). Variables describing maternal characteristics and infant food introduction were sourced, respectively, from interviews completed antenatally and during late infancy. The NZ Infant Feeding Guidelines were used to define early (≤ 4 months) and late (≥ 7 months) introduction. Associations were examined using multivariable multinomial regression, presented as adjusted relative risk ratios and 95 % confidence intervals (RRR; 95% CI). Complementary food introduction was early for 40·2 % and late for 3·2 %. The prevalence of early food group introduction were fruit/vegetables (23·8 %), breads/cereals (36·3 %), iron-rich foods (34·1 %) and of late were meat/meat alternatives (45·9 %), dairy products (46·2 %) and fruits/vegetables (9·9 %). Compared with infants with timely food introduction, risk of early food introduction was increased for infants: breastfed < 6months (2·52; 2·19–2·90), whose mothers were < 30 years old (1·69; 1·46–1·94), had a diploma/trade certificate *v.* tertiary education (1·39; 1·1–1·70), of Māori *v.* European ethnicity (1·40; 1·12–1·75) or smoked during pregnancy (1·88; 1·44–2·46). Risk of late food introduction decreased for infants breastfed < 6 months (0·47; 0.27–0·80) and increased for infants whose mothers had secondary *v.* tertiary education (2·04; 1·16–3·60) were of Asian *v.* European ethnicity (2·22; 1·35, 3·63) or did not attend childbirth preparation classes (2·23; 1·24–4·01). Non-timely food introduction, specifically early food introduction, is prevalent in NZ. Interventions to improve food introduction timeliness should be ethnic-specific and support longer breast-feeding.

as The first 1000 days of life, from conception to 2 years of age, are critical due to rapid growth and cognitive development and because nutrition in this period will have impact not only at that time but also throughout life^(1,2)^. Breast milk or infant formula alone supplies the nutritional needs of infants until the age of 6 months. After this age, the introduction of foods should be started to attend to an infant’s nutritional and developmental needs. This transition from only milk to other foods constitutes a critical period during early life^(3,4)^. In New Zealand (NZ), the Ministry of Health recommends that complementary feeding, defined as the gradual offering of other foods, solids or liquids, along with breast milk or infant formula, should be initiated around 6 months of age^(3)^. The NZ guidelines also state that complementary foods should include items from core food groups and that the first items to be introduced should be good sources of iron such as iron-fortified cereals, puréed vegetables, age-appropriate meats and vegetarian alternatives^(3)^.

The description of the timing of food introduction and its determinants within a population is important, since previous studies and systematic reviews suggest that non-timely food introductions are related to overweight/obesity among infants/toddlers which can persist across the lifespan^(5,6)^, gastrointestinal infections^(3,7,8)^, delayed growth and development^(8,9)^ and iron deficiency anaemia^(10)^. Gastrointestinal infections are related to early food introduction^(3,7,8,11,12)^, and impaired growth and development are related to late introduction (potentially due to shortage of vitamins and minerals such as iron, iron and vitamin A)^(8,13)^, while iron deficiency anaemia and child overweight/obesity are related to both, early and late food introduction^(6,10,14–18)^

The literature describing the relationship between the occurrence of food allergy and the timing of food introduction is controversial. For many decades, delaying the introduction of some foods was considered the most effective strategy to prevent food allergy^(19,20)^, but more recent research has demonstrated that such delay does not prevent the development of food allergy, with some research indicating that late food introduction may increase the risk of allergy^(21–23)^. Recent recommendations for the prevention of food allergy suggest that allergenic foods, such as peanuts, tree nuts, seeds, eggs, fish, shellfish, cows’ milk products, wheat and soyabeans, should be introduced around 6 months of age, alongside other recommended foods^(19,20,24–27)^.

Recently published information on infant feeding practices from 80 countries reported that only two-thirds of infants aged 6 to 8 months receive complementary foods, and that 15 % of infants are introduced to foods/drinks when aged between 2 and 3 months. Among 0–5-month-old infants, 43 % are given liquids or foods other than breast milk within the first 3 d of life^(28)^.

Previous studies have also reported inequities in the prevalence of non-timely food introduction, with infants whose mothers were younger, with lower levels of education and income, from minority ethnic groups, who smoked during pregnancy, or who had higher pre-gestational body mass index (BMI) being more likely to be exposed to non-timely food introduction^(29–34)^.

NZ currently does not have nationally generalisable or representative information on age of introduction of complementary feeding (defined as any food or drink other than breast milk or suitable infant formula milk) nor on associated maternal sociodemographic and health behaviour determinants. The only indicator collected routinely by the annual health surveys reports prevalence of introduction of solid foods before the 4th and before the 6th month of life (which is asked only to caregivers in households that have children aged 4 months to < 5 years)^(35)^. Findings from these annual surveys showed that, since 2006/2007, there has been no statistically significant change in the prevalence of infants who were introduced to solid foods before the 4th month of life. In 2019/20, 8·0 % of infants were introduced to solid foods before 4 months of age, with differences by sex (10·1 % for boys and 5·1 % for girls), maternal ethnicity (European/other: 6·9 %; Asian: 7·0 %; Māori: 13·8 % and Pacific: 15·1 %) and neighbourhood deprivation (5·1 % in the least deprived households and 11·7 % in the most deprived ones)^(35)^ Previous study, using data from a nationally generalisable birth cohort – *Growing Up in New Zealand* (*GUiNZ*), has also described the cohort’s age of introduction to solids, showing that 39·3 % were introduced to solids before or at the age of 4 months^(36)^.

In this study, using the *GUiNZ* cohort data, we aimed to examine (i) the prevalence of non-timely introduction (early and late) of complementary foods and of core food groups among 7–12-month-olds and (ii) the associations of maternal sociodemographic and health behaviour characteristics with infants’ non-timely food introduction.

## Methods

### Study population, data collection waves and ethical approval

We used data from the ongoing NZ birth cohort study, *GUiNZ*, which enrolled 6822 pregnant women and their 6853 children who formed the cohort. Eligibility was defined by maternal residency within a region of NZ chosen for its ethnic and socio-economic diversity of the birth cohort and mothers having an estimated delivery date between 25 April 2009 and 25 March 2010. This cohort has shown to be broadly generalisable to all NZ births from 2007 to 2010^(37,38)^. This investigation was conducted according to the guidelines laid down in the Declaration of Helsinki, and all procedures involving human subjects were approved by the Ministry of Health Northern Y Regional Ethics Committee (NTY/08/06/055). Written informed consent was obtained from all mothers/caregivers^(37,38)^.

Information was sourced from four data collection waves, antenatally (conducted mostly during the third trimester of pregnancy) and when infants were approximately 6 weeks, 9 and 31 months old. Variables describing maternal sociodemographic and health behaviours were sourced from the antenatal face-to-face computer-assisted personal interview, completed during the years of 2009/10. Infants’ perinatal information (sex, fetal count, birth and gestational age) and infants’ feeding status in the first weeks of life were obtained from perinatal data linkage and the 6-week computer-assisted telephone interview, conducted with each infant’s mother. Information on infant’s age of food introduction was obtained from the 9-month mother computer-assisted personal interview. Variables measuring the duration of any breast-feeding were derived from the 6-week computer-assisted telephone interview, 9-month computer-assisted personal interview and the 31-month computer-assisted telephone interview completed with each infant’s mother^(39)^.

**Fig. 1.**
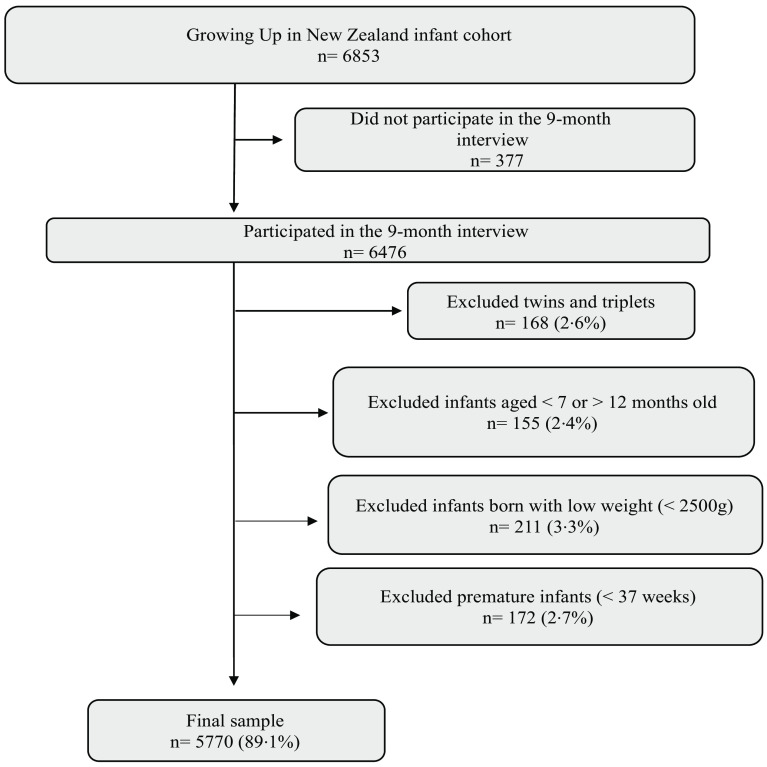
Flow chart describing the study sample.

Of the 6476 children whose mothers took part in the 9-month interview, we excluded 168 (2·6 %) twin/triplets, 211 (3·3 %) infants who were born with low birth weight (< 2500 g), 172 (2·7 %) with premature gestation (< 37 weeks of gestational age) and 155 (2·4 %) who were aged < 7 months or > 12 months at the time of the 9-month computer-assisted personal interview. As we examined the age of food introduction among infants, those > 12 months of age were excluded from the analyses. Infants aged < 7 months were excluded to ensure that every infant had the chance to have complementary feeding initiated and then their timing of food introduction classified as timely, early and late based on the national guidelines^(3)^. Twins/triplets were excluded so that only independent maternal observation was included. Children with birth weight less than 2500 grams (g) or gestational age less than 37 weeks were excluded because the NZ Infant Feeding Guidelines^(3)^ on timing of food introduction were established for healthy infants and may not be appropriate for pre-term or low birth weight infants, many of whom would be following tailored clinical nutrition guidelines. Thus, our final analytical sample was constituted of 5770 infants, corresponding to 89·1 % of the infants whose mother took part in the 9-month interview (Fig. 1).

### Outcomes

A 25-item semi-quantitative FFQ was used to assess the age of introduction of complementary feeding at the 9-month interview. Mothers were asked to report, retrospectively, the age (which was asked in rounded months) when their infants were introduced to the following foods: infant formula or milk other than breast milk; baby rice; baby breakfast cereal; other cereal; bread or toast; rusks; biscuits; vegetables – raw or cooked; fruits – fresh and canned; meat, chicken and meat dishes; fish and fish dishes – fresh and canned; eggs; milk puddings, rice pudding, yogurt and custards; nuts or peanut butter; shellfish; soya foods, tofu and soya desserts; sweets; chocolate; hot chips; potato chips – crisps; fruit juices – includes watered down juice; herbal drinks; tea; coffee; and soft drinks^(39)^. The FFQ was designed by an experienced academic paediatric dietitian, who selected items based on the NZ Infant Feeding Guidelines^(3)^ and foods and beverages commonly fed to NZ infants^(40,41)^.

The outcomes assessed in this study were infants’ age of introduction (in months) for (i) complementary foods and (ii) core food groups nationally recommended to infants (fruits and vegetables; meats and alternatives; breads and cereals; dairy foods and; iron-rich foods)^(3)^. Age when complementary feeding started was defined as the age when infants first tried any foods or drinks other than breast milk and suitable infant formula^(3)^. The age of introduction of each core food group was defined as the age when infants first tried any food or drink belonging to each core food group. The list of foods considered under each core food group is provided in Table 1. Following the NZ Infant Feeding Guidelines^(3)^, food introduction was classified as early if introduced ≤ 4 months of age, timely if introduced when infants were 5–6 months of age and late if introduced ≥ 7 months of age.

**Table 1.** Food items with the *Growing Up in New Zealand* cohort study FFQ included in each of the core food groups recommended for infants

Food groups and other foods recommended^(3)^	Food items from the FFQ that were included
Fruits and vegetables	Fruits (includes fresh and canned) and vegetables (raw or cooked).
Breads and cereals	Baby rice, baby breakfast cereal, other cereal, breads or toast, rusks and biscuits.
Dairy foods	Milk puddings, rice pudding, yogurt and custards.
Meat and alternatives	Meat, chicken, meat dishes, fish, fish dishes (includes fresh and canned), eggs, nuts or peanut butter, shellfish, soya foods, tofu, and soya desserts.
Iron-rich foods	Meat, chicken, baby rice and baby breakfast cereal.

### Covariates

Informed by the published literature, we examined the potential influence of antenatal maternal sociodemographic and health behaviour factors on the timing of infant food introduction^(29–34,42)^.

The following antenatal maternal sociodemographic factors were examined: parity, pregnancy planning, level of education completed, age, maternal ethnicity and socio-economic status. Maternal ethnicity was self-prioritised and reported at the Statistics NZ Level 1 classification. The ethnic groupings used in this study were (1) European, (2) Māori, (3) Pacific Peoples, (4) Asian, (5) Middle Eastern, Latin American and African (MELAA), and (6) Other^(43–45)^. The groups MELAA and Other were combined for analysis purposes. Socio-economic status was described using the NZDep2006 Index of Deprivation^(35)^. This validated small area measure of neighbourhood deprivation combines nine socio-economic characteristics from the 2006 NZ census data collected at aggregations of approximately 100 people and assigned to individual households based on geo-coded address data^(43)^. NZDep2006 scores are ranked in deciles where deciles 1–2 represent the least deprived neighbourhoods and deciles 9–10 represent the most deprived ones.

The antenatal maternal health behaviour characteristics examined were: adherence to nutrition guidelines during pregnancy, pre-pregnancy BMI, smoking patterns before/during pregnancy, physical activity (PA) before/during pregnancy and mothers’ attendance at childbirth preparation classes. During pregnancy, the assessment of the adequacy of the number of servings consumed of vegetables and fruit, breads and cereals, milk and milk products and, lean meat, meat and alternatives and eggs was based on the national recommendations for pregnant women that were in place when the antenatal interview took place^(46)^. For each food group, daily intakes were classified as ‘yes’ (≥ number of servings recommended) and ‘no’ (< number of servings recommended)^(44)^. Pre-pregnancy BMI was calculated based on self-reported weight and height and classified according to the WHO cut-offs^(47)^. Smoking patterns pre-/during pregnancy were categorised as continued smoking, stopped smoking during pregnancy and non-smokers. PA before or during pregnancy was estimated using the International Physical Activity Questionnaire (IPAQ)^(40)^. Women who engaged in moderate PA for at least 30 minutes for at least 5 out of 7 d, or vigorous PA for at least 30 minutes on at least 2 out of 7 d were classified as participating in moderate/vigorous activity. The PA categories examined were: no moderate/vigorous PA before or during pregnancy, moderate/vigorous PA before and during pregnancy, and moderate/vigorous PA only before or only during pregnancy^(48)^. Mother’s attendance of childbirth preparation classes was classified as: yes; no, but intend to attend and; and no, and no intention to attend. Antenatal classes are provided by non-governmental organisations during pregnancy, and parents are recommended to participate during each pregnancy or at least during the first pregnancy^(49)^. The main purpose of these classes is to provide information about a variety of pregnancy and childbirth-related issues including: how the NZ maternity system works; pregnancy care; normal changes in maternal health that occur during pregnancy; healthy pregnancy; labour and childbirth; postnatal care; breast-feeding and safe sleeping^(50)^.

We estimated the duration of any breast-feeding (in months) according to methodology described in Castro *et al.* ^(38)^, which was categorised as < 6 months and ≥ 6 months of duration for analytical purposes.

### Statistical analyses

Categorical variables were described as proportions and continuous variables as medians and interquartile ranges. The prevalence of timely and non-timely (early and late) age of food introduction was calculated. Infants who were never introduced to complementary feeding or who never tried items under the core food groups by the date of the 9-month interview were included in the denominators. We used *χ* ^2^ tests to compare proportions of infants introduced early and late to foods according to infant sex and breast-feeding duration.

It was examined the associations between the timing of introduction to complementary feeding [dependent variable categorised as timely (0), early (1) or late (2)] and the maternal characteristics (independent variables). Relative risk ratios (RRR) and 95% confidence intervals (95% CI) were obtained from univariable and multivariable multinomial logistic regression models. Univariable associations with *P* < 0·15 were used to identify the independent variables to be tested in the multivariable models, following a forward stepwise approach. Variables were retained in the final model if associations with the dependent variable had *P* < 0·05 or if they changed the magnitude of the RRRs by 10 % or more. Infant sex (boy/girl), age (in months-continuous variable) and birth weight (in g-continuous variable) adjusted the final model as these variables are potentially important factors influencing the timing of food introduction^(30,31)^.

All analyses were performed using Stata Statistical Software (STATA) (version 14, StataCorp. 2015. Stata Statistical Software: Release 14. StataCorp LP).

## Results

### Prevalence of early and late introduction of complementary feeding and of core food groups (overall, by sex and by breast-feeding duration)

**Fig. 2.**
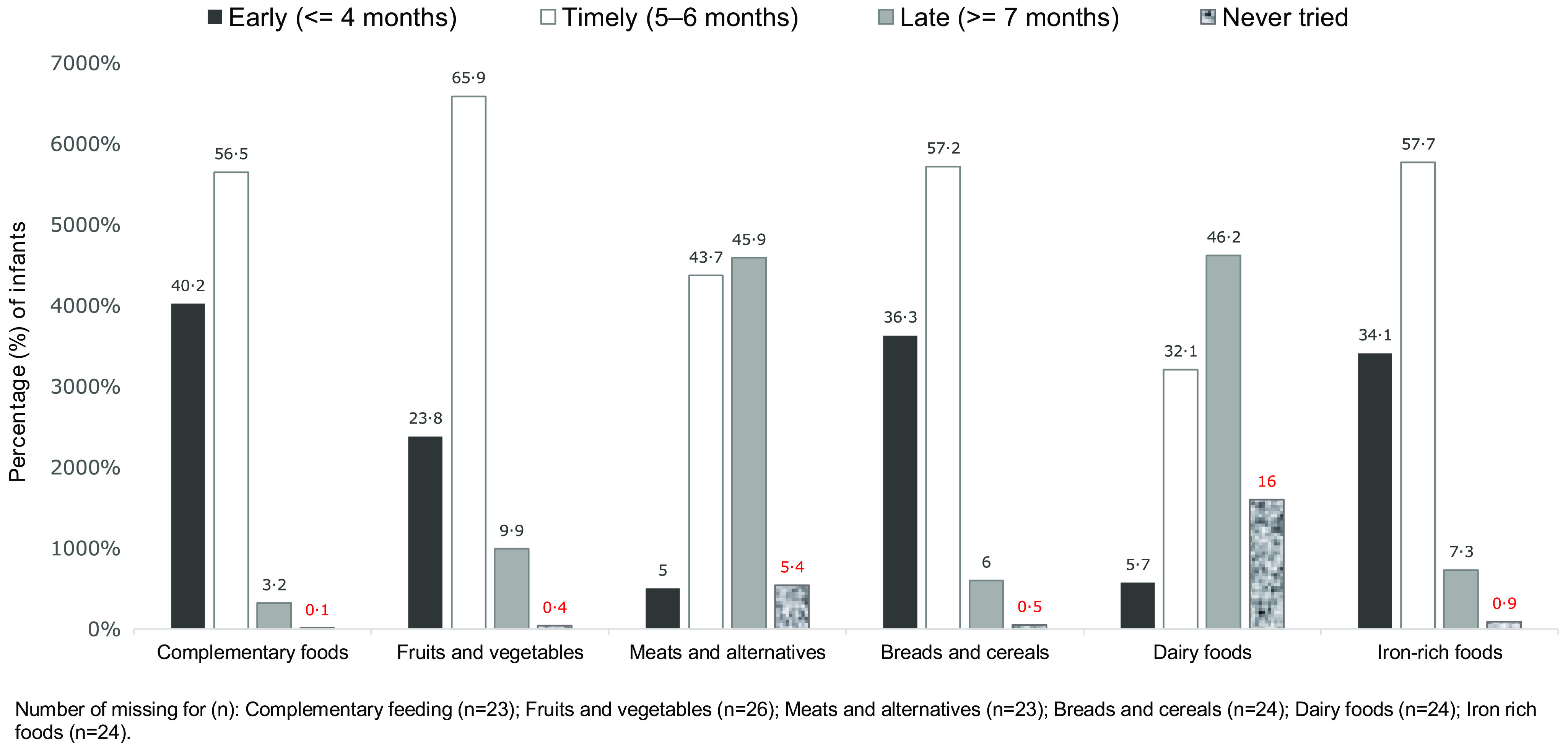
Prevalence of timely (5–6 months), early (< 4 months) and late (> 7 months) introduction of complementary feeding and of each recommended core food group (all cohort, *n* = 5770), *Growing Up in New Zealand cohort*.

Four in ten infants were introduced to complementary feeding early (age ≤ 4 months), and 3·2 were introduced to complementary feeding late (age ≥ 7 months). The prevalence of early introduction for core foods groups were: breads and cereals (36·3 % of infants), iron-rich foods (34·1 %), and fruit and vegetables (23·8 %). The prevalence of late introduction for core food groups were: meats and alternatives (45·9 % of infants), dairy foods (46·2 %), and fruits and vegetables (9·9 %) (Fig. 2). By the date of the 9-month interview, the proportion of the infants who were not introduced to complementary feeding or to the core food groups were: complementary foods (0·05 %), fruits and vegetables (0·4 %), meat and alternatives (5·36 %), breads and cereals (0·54 %), dairy products (16·05 %) and iron-rich foods (0·89 %).

When comparing boys and girls, there were no statistically significant differences in the prevalence of early and late introduction of complementary feeding and of the core food groups (data not shown in figure).

**Fig. 3.**
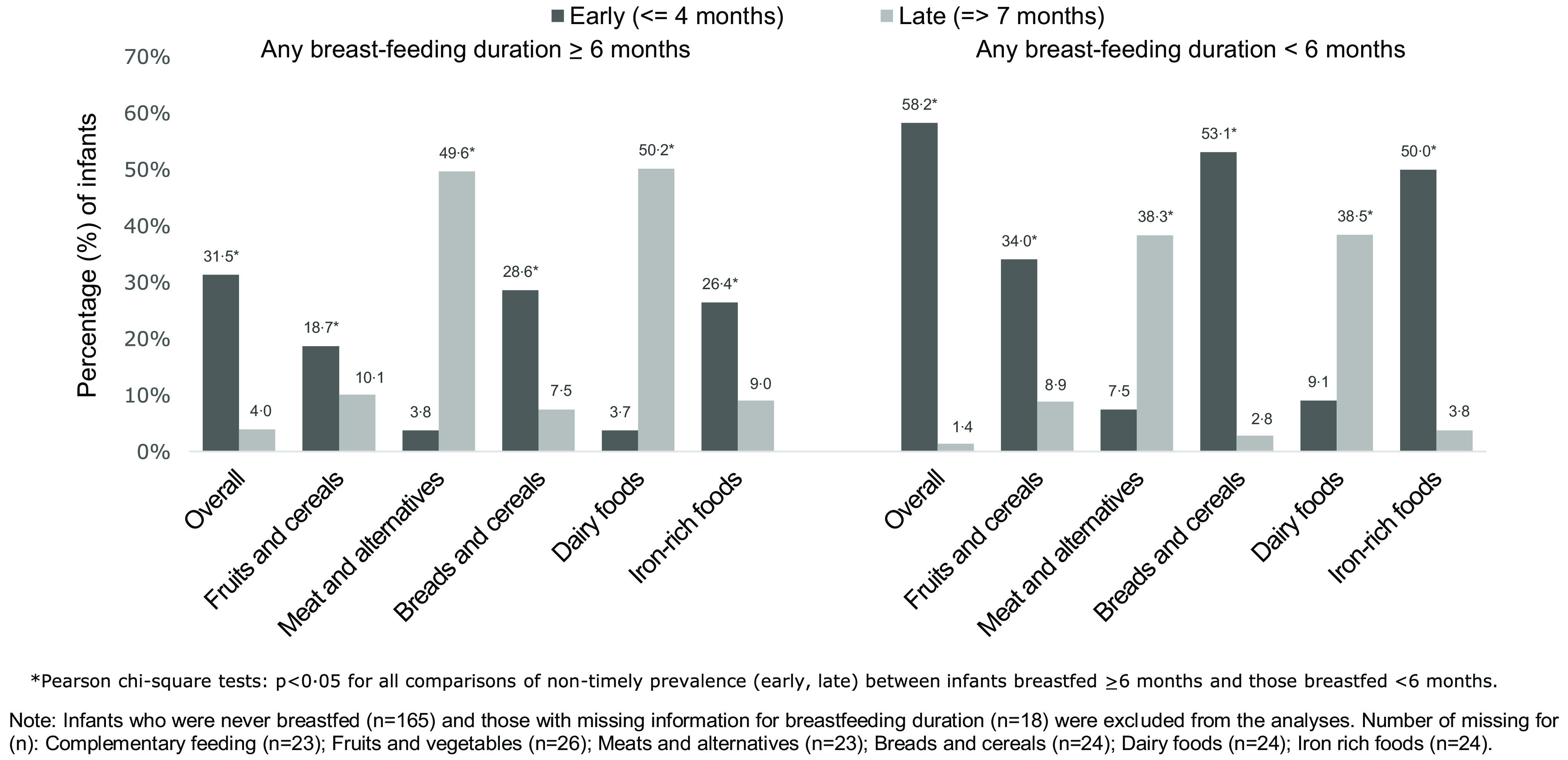
Prevalence of early (< 4 months) and late (> 7 months) introduction of complementary feeding and of each recommended core food group according to duration of any breast-feeding (< 6 months: *n* 1740; ≥ 6 months: *n* 3847), *Growing Up in New Zealand* cohort.

Figure 3 displays the prevalence of early and late food introduction, according to infants’ duration of any breast-feeding. Overall and for each core food group, the prevalence of early introduction of complementary feeding and introduction of breads and cereals, Fe-rich foods, and fruits and vegetables were significantly higher among infants breastfed for < 6 months in comparison with infants breastfed for ≥ 6 months. In contrast, the prevalence of late introduction of dairy products and meat and alternatives were significantly higher among infants breastfed for ≥ 6 months in comparison with infants breastfed for < 6 months (Fig. 3).

### Prevalence of early and late introduction of complementary feeding according to infant’s and mothers’ characteristics

The median (interquartile ranges) age of the children who had timely, early and late introduction to complementary feeding were, respectively, 8·80 (8·61–9·01), 8·84 (8·61–9·20) and 9·00 (8·64–9·43) months. Children who had timely, early and late introduction to complementary feeding had median (interquartile ranges) birth weights of 3540 (3250–3860), 3580 (3270–3890) and 3600 (3280–3900) g (data not shown in table).

A larger proportion of infants who had early and late introduction to complementary foods were male (early: 53·6 % and late: 53·6 %), breastfed to ≥ 6 months (early: 52·1 % and late: 83·9 %) or from planned pregnancies (early: 54·3 % and late: 51·9 %). A larger proportion of their mothers were multiparous (early: 56·5 % and late: 71·8 %), non-smokers (early: 71·7 % and late: 86·3 %), with educational level lower than bachelor’s degree (early: 70·6 % and late: 73·2 %), from neighbourhoods with deprivation deciles 7–10 in the NZDep2006 index (early: 53·8 % and late: 62·9 %) or either did not attend and did not intend to attend childbirth preparation classes (early: 59·6 % and late: 77·1 %). The majority of infants’ mothers did not adhere to national recommended intakes of vegetables and fruit (early: 76·2 % and late 74·4 %), breads and cereals (early: 69·5 % and late: 76·7 %) and lean meat, meat alternatives and eggs (early: 76·6 % and late: 71·2 %) during pregnancy. The majority of infants’ mothers adhered to national recommended intakes of milk and milk products in pregnancy (early: 58·8 % and late: 53·8 %). Among the mothers who introduced complementary feeding early to their infants, 92·9 % were ≥ 20 years old, 49·5 % were of European ethnicity and for 54·4 % pre-pregnancy BMI were > 25 kg/m^2^. Among the mothers who introduced complementary feeding late to their infants, 56·9 % were ≥ 30 years of age, 34·8 % were of European ethnicity, and for 44 % pre-pregnancy BMI were > 25 kg/m^2^ (Table 2).

**Table 2.** Prevalence of early, timely and late introduction of complementary feeding according to infant and maternal characteristics (number and percentages)

Infant’s and mother’s characteristics*	Timing of food introduction					
	Early (≤ 4 months)		Timely (5–6 months)		Late (≥ 7 months)	
	*n*	%	*n*	%	*n*	%
Infants						
Sex (*n* 5744)						
Male	1238	53·6	1639	50·4	97	53·6
Female	1074	46·4	1612	49·6	84	46·4
Breast-feeding duration (*n* 5726)						
≥ 6 months	1199	52·1	2474	76·3	151	83·9
< 6 months	1013	44·0	701	21·6	25	13·9
Never breastfed	91	3·9	68	2·1	< 10†	
Mothers						
Parity (*n* 5730)						
Primiparous	1001	43·5	1345	41·4	51	28·2
Multiparous	1301	56·5	1902	58·6	130	71·8
Pregnancy planned (*n* 5708)						
Yes	1245	54·3	2202	68·1	94	51·9
No	1048	45·7	1032	31·9	87	48·1
Attendance to childbirth preparation class (*n* 5689)						
Yes	478	20·9	754	23·4	16	8·9
No but intend to attend	445	19·5	576	17·9	25	14·0
No and do not intend to attend	1362	59·6	1895	58·7	138	77·1
Age (years) (*n* 5735)						
< 20	165	7·1	92	2·8	< 10†	
20–29	1096	47·6	1034	31·8	75	41·4
≥ 30	1043	45·3	2124	65·4	103	56·9
Ethnic group (*n* 5725)						
European	1138	49·5	1968	60·7	63	34·8
Māori	437	19·0	302	9·3	24	13·3
Pacific	359	15·6	377	11·6	51	28·2
Asian	288	12·5	484	15·0	38	21·0
MELAA and Other	79	3·4	112	3·4	< 10†	
Level of education (*n* 5720)						
No secondary school qualification	223	9·7	129	4·0	14	7·8
Secondary school/NCEA 1–4	575	25·0	673	20·7	70	39·1
Diploma/trade cert/NCEA 5–6	824	35·9	881	27·2	47	26·3
Bachelor’s degree	406	17·7	916	28·1	26	14·5
Higher than bachelor’s degree	268	11·7	646	20·0	22	12·3
NZDep 2006 (deciles)** (*n* 5733)						
1–2 (least deprived)	298	12·9	630	19·4	19	10·5
3–4	380	16·5	687	21·1	24	13·3
5–6	386	16·8	581	17·9	24	13·3
7–8	519	22·5	646	19·9	36	19·8
9–10 (most deprived)	720	31·3	705	21·7	78	43·1
Pre-pregnancy BMI (kg/m^2^) (*n* 5072)						
< 25	1071	54·6	1874	63·3	84	56·0
≥ 25–< 30	458	23·3	632	21·4	36	24·0
≥ 30	434	22·1	453	15·3	30	20·0
Physical activity before and during pregnancy (*n* 5260)						
Moderate/vigorous activity before and during pregnancy	747	35·2	975	32·7	61	38·1
Moderate/vigorous activity only before or during pregnancy	554	26·1	812	27·3	38	23·8
No moderate/vigorous activity before and during pregnancy	820	38·7	1192	40·0	61	38·1
Pre-/during pregnancy smoking pattern (*n* 5245)						
Non-smoker	1514	71·7	2582	86·9	138	86·3
Stopped smoking	273	12·9	232	7·8	12	7·5
Continued smoking	325	15·4	159	5·3	10	6·2
Fruits and vegetables guideline adherence (*n* 5260)						
Yes	505	23·8	767	25·8	41	25·6
No	1616	76·2	2212	74·2	119	74·4
Breads and cereals guideline adherence (*n* 5260)						
Yes	647	30·5	693	23·3	49	30·6
No	1474	69·5	2286	76·7	111	69·4
Milk and derivatives guideline adherence (*n* 5260)						
Yes	1246	58·8	1729	58·0	86	53·8
No	875	41·2	1250	42·0	74	46·2
Meat and alternatives guideline adherence (*n* 5258)						
Yes	495	23·3	608	20·4	46	28·8
No	1626	76·7	2369	79·6	114	71·2

NCEA, National Certificate of Education Achievement; MELAA, Middle Eastern, Latin American and African; NZDep2006, neighbourhood deprivation index 2006; *kg/m* ^*2*^, kilograms/squared metre.

Infants aged 7 to 12 months in the 9-month interview.

* Missing for variables listed (*n*): sex (*n* 26); breast-feeding (*n* 44); parity (*n* 40); pregnancy planned (*n* 62); childbirth preparation class (*n* 81); age group (*n* 35); ethnicity (*n* 45); education (*n* 50); deprivation index (*n* 37); BMI pre-pregnancy (*n* 698); physical activity (*n* 510); smoking pattern (*n* 525); adherence to fruits and vegetables consumption (*n* 510); adherence to breads and cereals consumption *n* 510; adherence to milk and derivatives consumption (*n* 510); and adherence to meats and alternatives consumption (*n* 512).

** Area-level socio-economic deprivation was measured by using the NZ Index of Deprivation, derived from the 2006 national census according to the methodology described in Salmond et al.^(34)^. NZDep2006 deciles 1–2 represent the least deprived neighbourhoods and deciles 9–10 the most deprived neighbourhoods.

† As per *Growing up in New Zealand* study anonymity requirement, ‘< 10’ represents greater than zero and less than ten children in the cell.

### Maternal sociodemographic and health behaviour characteristics associated with early and late food introduction

In univariable analyses, except for mother’s adherence to recommended intakes of milk and milk derivatives during pregnancy, all mothers’ sociodemographic and health behaviour characteristics were associated with timing of food introduction (*P* < 0·15) and were therefore tested in the multivariable models (online Supplementary Table S1).

The results of the multivariable analysis showing the variables retained in the final model are presented in Table 3. When compared with infants with timely introduction of complementary foods, the risk of being introduced to foods early was increased for those infants who were never breastfed or were breastfed for < 6 months (*v*. breastfed for ≥ 6 months), whose mothers were younger than 30 years of age (*v*. *≥* 30 years*)*, who had a diploma/trade certificate (*v*. a higher tertiary educational qualification), of Māori ethnicity (*v*. European ethnicity) or, who continued smoking during pregnancy (*v*. non-smokers). When compared with infants with timely food introduction, the risk of late introduction of food was decreased for infants who were breastfed for < 6 months (*v*. breastfed for ≥ 6 months) and was increased for infants whose mothers had secondary school-level education (*v*. a higher tertiary educational qualification), of Asian ethnicity (*v*. European ethnicity), or who did not attend nor had the intention to attend childbirth preparation classes (*v*. mothers who attended childbirth preparation classes).

**Table 3.** Adjusted RRR and 95 % CI for the associations between timing of food introduction and variables describing breast-feeding duration and maternal sociodemographic and health behaviours (all cohort, *n* = 4595)

Infant’s and mother’s characteristics	Age of introduction of complementary feeding*,**					
	Early (≤ 4 months)			Late (≥ 7 months)		
	Adjusted RRR	95 % CI	*P*	Adjusted RRR	95 % CI	*P*
Breast-feeding duration						
≥ 6 months	1·00	Ref.		1·00	Ref.	
< 6 months	2·52	2·19, 2·90	< 0·001	0·47	0·27, 0·80	0·006
Never breastfed	2·00	1·34, 2·99	0·001	0·79	0·24, 2·64	0·708
Age (years)						
≥ 30	1·00	Ref.		1·00	Ref.	
20–29	1·69	1·46, 1·94	< 0·001	0·98	0·65, 1·46	0·904
≥ 30	2·37	1·60, 3·53	< 0·001	0·59	0·13, 2·60	0·482
Attendance to childbirth preparation class						
Yes	1·00	Ref.		1·00	Ref.	
No but intend to attend	1·05	0·86, 1·28	0·619	1·69	0·85, 3·37	0·133
No and do not intend to attend	1·02	0·87, 1·21	0·774	2·23	1·24, 4·01	0·008
Level of education						
Higher than bachelor’s degree	1·00	Ref.		1·00	Ref.	
Bachelor’s degree	0·90	0·74, 1·11	0·331	0·64	0·35, 1·18	0·153
Diploma/trade cert/NCEA 5–6	1·39	1·14, 1·70	0·001	1·05	0·59, 1·87	0·877
Secondary school/NCEA 1–4	1·14	0·91, 1·42	0·258	2·04	1·16, 3·60	0·013
No secondary school qualification	1·19	0·82, 1·73	0·364	1·77	0·66, 4·71	0·254
Self-prioritised ethnic group						
European	1·00	Ref.		1·00	Ref.	
Māori	1·40	1·12, 1·75	0·003	1·38	0·72, 2·61	0·329
Pacific	1·08	0·84, 1·39	0·557	1·44	0·75, 2·76	0·275
Asian	1·09	0·90, 1·33	0·377	2·22	1·35, 3·63	0·002
MELAA and Other	1·25	0·89, 1·76	0·196	1·35	0·52, 3·50	0·542
Pre-/during pregnancy smoking pattern						
Non-smoker	1·00	Ref.		1·00	Ref.	
Stopped smoking	1·22	0·97, 1·53	0·094	0·71	0·33, 1·52	0·377
Continued smoking	1·88	1·44, 2·46	< 0·001	0·79	0·33, 1·85	0·580

Ref., reference category; RRR, relative risk ratio; NCEA, National Certificate of Education Achievement; MELAA, Middle Eastern, Latin American and African.

Missing for variables listed in the table (*n*): sex (*n* 26); age in years (*n* 26); birth weight (*n* 26); breast-feeding (*n* 44); parity (*n* 40); planned pregnancy (*n* 62); age group (*n* 35); childbirth preparation class (*n* 81); education (*n* 50); ethnicity (*n* 45); deprivation index (*n* 37); BMI pre-pregnancy (*n* 698); physical activity (*n* 510); smoking pattern (*n* 525); adherence to fruits and vegetables consumption (*n* 510); adherence to breads and cereals consumption *n* 510; adherence to milk and derivatives consumption (*n* 510); and adherence to meats and alternatives consumption (*n* 512).

* Infants singletons aged 7–12 months at the 9-month interview.

** The group of mothers whose infants had timely introduction to complementary foods was the reference group in these analyses.

Multivariable multinominal logistic regression model included all variables presented in the table adjusted by maternal adherence to bread and cereals guideline, pre-pregnancy BMI, NZDep 2006***, pregnancy planned, child’s birth weight, sex and age (in months).

*** Area-level socio-economic deprivation was measured by using the NZ Index of Deprivation, derived from the 2006 national census according to the methodology described in Salmond et al.^(34)^. NZDep2006 deciles 1–2 represent the least deprived neighbourhoods and deciles 9–10 the most deprived neighbourhoods.

## Discussion

### Summary of findings

This nationally generalisable birth cohort showed that 40·2 % and 3·2 % of infants were introduced to complementary feeding early and late, respectively. Breads and cereals, iron-rich foods, and fruit and vegetables were introduced early to, respectively, 36·3 %, 34·1 % and 23·8 % of the infants. Meat and alternatives and dairy products were introduced late to 45·9 % and fruits and vegetables to 9·9 % of the infants. There were no significant sex differences in the prevalence of non-timely introduction of complementary feeding and of the core food groups. Early introduction of complementary feeding was more prevalent among infants breastfed for < 6 months (*v*. ≥ 6 months), and late introduction of complementary feeding was more prevalent among infants breastfed for ≥ 6 months (*v*. < 6 months). In the multivariable model, non-timely food introduction was independently associated with breast-feeding duration and with maternal attendance/intention to attend childbirth preparation classes, education level, self-prioritised ethnicity, age and smoking patterns during pregnancy.

### Comparisons of findings with previous studies

Challenges to comparing the prevalence of early, timely and late introduction of complementary feeding in our study with previous investigations include the diversity of indicators of timing of food introduction used (e.g. introduction of complementary feeding, introduction to solids and introduction to core food groups). In addition, the cut-offs used by different studies to classify food introduction as timely vary widely. The high prevalence of early introduction of complementary feeding within the *GUiNZ* cohort born in 2009/2010 (40·2 %) was concerning and significantly higher than prevalence reported for population-based studies performed in the Netherlands in 2009/2010 (21·4 %)^(51)^, in the USA in 2009–2014 (16·3 %)^(52)^, and in Poland and Austria in 2017–2019 (3 %)^(53)^. The prevalence of early complementary feeding within the *GUiNZ* cohort was also higher than the prevalence in longitudinal Australian studies reporting data on early introduction of any foods from core food groups (12 %)^(31)^, of soft, semi-solid and solid foods (33·3 %)^(54)^, and of solid foods (20·0 %)^(55)^. However, the prevalence of late introduction of complementary feeding within the *GUiNZ* cohort (3·2 %) was similar or lower to prevalence reported for Australian cohorts [3 %(43); 10 %(44)] and for the infants in the Dutch^(51)^, American^(52)^, Polish^(53)^ and Austrian^(53)^ population (2 %, 12·9 %, 37·1 % and 20·4 %, respectively).

Particularly concerning aspects regarding the introduction of core food groups in our study were the late introduction of iron-rich foods (7·3 %) and meat and alternatives (45·9 %), as foods in these food groups are recommended to be the first ones introduced due to their high iron content^(3)^. Another concerning finding from this study is that one in ten infants had late introduction to fruits and vegetables. A cross-sectional study conducted in London nursery schools found that the earlier the introduction of fruits and vegetables, the higher the intake of these items among 2–6-year-olds^(56)^. However, a prospective study using data from four European cohorts found that associations between age of introduction to fruits and vegetables and intake of fruits and vegetables at the pre-school years were weaker and less consistent across the cohorts^(57)^.

We did not find significant associations between infants’ sex and early food introduction, similar to some studies^(58,59)^ but contrary to others^(60–63)^ which reported that boys were more likely to be introduced early to foods. Findings from the NZ health survey in 2019/20 for the indicator of introduction of solid foods before the age of 4 months also showed that boys were twice as likely as girls to be introduced early to solids^(35)^. A systematic review reported that mothers’ reasons for introducing foods earlier to boys are multiple and include, among others, the mother’s perception of their infant’s needs^(29)^.

The higher prevalence of early food introduction among infants who were never breastfed or who were breastfed for shorter periods has also been reported in previous studies from the Netherlands, USA, Austria and Poland^(51–53)^. It is well established that breast milk intake is displaced by infant formula milk, however, until recently, it was not clear if introduction of solid foods has the same effect in the duration of breast-feeding^(64)^. Lessa *et al.* ^(64)^, using data from three large representative UK cohorts completed in the last 25 years, identified that, independent of background characteristics, earlier introduction of solids was associated with a shorter duration of breast-feeding with a dose–response relationship, especially in cohorts where earlier introduction of solids was the norm. A NZ trial (BLISS) which assessed the impact of a baby-led approach to complementary feeding (involving the recommendation of delaying the introduction of complementary feeding to the age of 6 months) found that BLISS infants were exclusively breastfed for longer than the control group^(65)^. The findings of Lessa et al^(64)^ and Taylor *et al.* ^(65)^ support the recommendation that strategies which promote timely introduction of foods, per se, may impact on longer duration of breast-feeding in NZ. This indicator was previously reported as falling well below national recommendations (where only 37 % of NZ infants were breastfed for ≥ 12 months and 12·5 % for ≥ 24 months^(39)^).

Similar to our findings, previous studies across Europe and Oceania^(30,51,53,66,67)^ reported that younger and less educated mothers were more likely to introduce foods early to their infants when compared with older and more educated mothers. Our study also showed that in relation to infants of European mothers, infants of Māori mothers were more likely to have early or late introduction to complementary feeding, while infants of Asian, Pacific and MELAA mothers were at increased risk of having late introduction to foods. Ethnic disparities in early-life nutrition have also been previously described within the *GUiNZ* cohort^(39,42,68)^, indicating that, to ensure adequate nutrition in the first 1000 d of life in NZ, it is vital to address cultural differences in feeding practices. The association between maternal smoking during pregnancy and early introduction of infants to complementary foods reported in our study and in previous studies^(30,60,69)^ may originate from behavioural, psychosocial and biological factors, such as observations that smoking mothers are less likely be health conscious and more likely to experience stress and have less social support and conscientiousness^(70)^.

Despite the independent association between late food introduction and attendance at childbirth preparation classes in NZ verified in our study, there is potential that this association may have been confounded or related to the unmeasured characteristics of parents who attend these classes, for example, health education seeking, and likelihood of following public health advice, among others. Additionally, the current content of childbirth classes in NZ appears to be heterogeneous, since some classes do not include content on infant feeding and timing of introduction to foods^(71)^.

### Strengths and limitations of the study

To the best of our knowledge, this represents the first NZ nationally generalisable study on the prevalence of non-timely food introduction and their main maternal determinants. The main limitation of this study refers to the level of accuracy of the reported prevalence of timely, early and late introduction given the following reasons. *GUiNZ* study did not collect information on infants’ age of introduction of water and cows’ milk. Each infant’s mother was only asked if their infant was introduced to cows’ milk by the date of the 9-month interview but not the age when the infants were introduced to it. Thus, the prevalence of early introduction of complementary feeding within the cohort may be potentially underestimated. However, the proportion of babies introduced to cows’ milk by the time of the 9-month interview was low within the cohort (2 % – data not presented). Another aspect is the fact that only one aggregated list of dairy foods (milk pudding, rice pudding, yogurt and custard) was included under the food group ‘dairy products’. Therefore, the lack of information on age of introduction of other recommended dairy products, such as cheese, may have potentially resulted in an overestimation of the prevalence of late introduction of dairy foods within the cohort. When calculating the prevalence of early, timely and late introduction, we opted to include those infants who were not introduced to complementary feeding or to the core food groups examined, in the denominators rather than rolling them to the group of late introduction. This is because we cannot assume that, for example, infants who were not introduced to dairy products or meats by the 9-month interview were introduced to these foods later in life. There may be situations where, for many reasons (health, religion and beliefs), parents may opt to not offer these items to their children. Finally, as the age of food introduction was retrospectively reported by the mothers only in months (and not in months and days), there is potential for inaccuracies in the estimation of the prevalence of early, timely and late food introduction within the *GUiNZ* cohort.

### Importance of findings to infant public health nutrition

The findings reported in this nationally generalisable study can be used to guide NZ food and nutrition policies that aim to improve and support optimal timing of food introduction in NZ. Ensuring appropriate nutrition in the first year of life represents one of the internationally recommended platforms to maximise the impact for the prevention and reduction of the double burden of malnutrition during childhood and throughout the life course^(72,73)^.

As previously described, non-timely food introduction is associated with relevant health and nutritional issues during infancy and also subsequently during childhood and later in life^(3,5–18)^. These include iron deficiency anaemia, food allergies and childhood obesity^(6,10,14–23)^, all child health conditions of public health relevance in NZ^(28)^. Iron deficiency is of concern, with 14 % of children aged 6–23 months affected in a population-based study conducted in Auckland (NZ) from 1999 to 2002^(74)^. Findings from the largest epidemiological study among children, the International Study on Asthma and Allergies in Childhood (ISAAC)^(75)^, found that NZ was rated in the ‘top five countries’ for asthma prevalence with levels similar to Australia, the UK, Ireland, the USA and Canada. It is assumed therefore that food allergy rates in NZ may be similar to rates found in those countries^(75)^. Based on findings from an Australian population-based study of 12-month-olds in 2011, it is estimated that food allergies may affect more than 10 % of NZ infants^(76)^. Childhood obesity represents one of the main national challenges in NZ, a country which was recently positioned second in the ranking for the prevalence of child/adolescent overweigh/obesity among the countries of the Organisation for Economic Cooperation and Development/European Union (39·5 % of 5–19-year-old children)^(28)^.

### Conclusions

Our findings suggest that interventions to increase the prevalence of timely food introduction in NZ should be culturally sensitive and encompass the promotion and support of breast-feeding duration according to national recommendations^(3)^, alongside improvements in policy and maternal health education. Future investigations will examine the impact of non-timely complementary feeding introduction on child/youth health and wellbeing outcomes in this cohort.

## Supplementary material

For supplementary material accompanying this paper visit https://doi.org/10.1017/S000711452200112X. click here to view supplementary material
