# Supplementary material for: Prevalence and maternal determinants of early and late introduction of complementary foods: results from the Growing Up in New Zealand cohort study
Source: Br J Nutr. 2022 Apr 11;129(3):491–502. doi: 10.1017/S000711452200112X (PMC9876814; doi:10.1017/S000711452200112X)
Supplement: Supplementary file 1 [file S000711452200112Xsup001.docx]

**Table S1.** Unadjusted relative risk ratios (RRR) and 95% confidence intervals (IC) for the associations between timing of food introduction and variables describing breastfeeding duration and maternal sociodemographic and health behaviours.

| **Antenatal maternal characteristics** | **N (%)*** | **Age of introduction of complementary feeding**** | | | | | |
| --- | --- | --- | --- | --- | --- | --- | --- |
|  |  | **Early introduction (age ≤ 4 months)** | | | **Late introduction (age ≥ 7 months)** | | |
|  |  | **Unadjusted RRR ^*^** | **(95% CI)** | **P value** | **Unadjusted RRR ^*^** | **(95% CI)** | **P value** |
| *Age in years (continuous)* | 5744 | 1.12 | (1.03-1.23) | 0.008 | 1.65 | (1.35-2.02) | 0.008 |
| *Birth weight (continuous)* | 5744 | 1.00 |  | 0.015 | 1.00 | (0.99-1.00) | 0.412 |
| *Sex*  Male  Female | 2974 (52.0)  2770 (48.0) | Ref  0.88 | (0.79-0.98) | 0.021 | Ref  0.88 | (0.65-1.19) | 0.406 |
| *Breastfeeding duration*  >6 months  <6 months  Never breastfed | 3824 (66.8)  1739 (30.4)  163 (2.8) | Ref  2.98  2.76 | (2.65-3.36)  (2.00-3.80) | <0.001  <0.001 | Ref  0.58  0.96 | (0.38-0.90)  (0.35-2.68) | 0.015  0.944 |
| *Parity*  Primiparous  Multiparous | 2397 (41.8)  3333 (58.2) | Ref  0.92 | (0.82-1.02) | 0.126 | Ref  1.80 | (1.29-2.51) | <0.001 |
| *Planned pregnancy*  Yes  No | 3541 (62.0)  2167 (38.0) | Ref  1.80 | (1.61-2.01) | <0.001 | Ref  1.97 | 1.46-2.67 | <0.001 |
| *Age group (years)*  >30 years  20-29 years  <20 years | 3270 (57.0)  2205 (38.4)  260 (4.6) | Ref  2.16  3.65 | (1.93-2.42)  (2.80-4.76) | <0.001  <0.001 | Ref  1.50  0.67 | (1.10-2.03)  (0.21-2.16) | 0.010  0.505 |
| *Attendance to childbirth preparation class*  Yes  No, but intend to attend  No, and do not intend to attend | 1248 (21.9)  1046 (18.4)  3395 (59.7) | Ref  1.22  1.13 | (1.02-1.44)  (0.99-1.30) | 0.022  0.067 | Ref  2.05  3.43 | (1.08-3.87)  (2.03-5.80) | 0.028  <0.001 |
| *Highest level of education*  Higher than bachelor’s degree  Bachelor’s degree  Diploma/Trade cert/NCEA 5-6  Secondary school/NCEA 1-4  No secondary school qualification | 936 (16.4)  1348 (23.6)  1752 (30.6)  1318 (23.0)  366 (6.4) | Ref  1.07  2.25  2.06  4.17 | (0.89-1.28)  (1.90-2.68)  (1.72-2.47)  (3.21-5.40) | 0.482  <0.001  <0.001  <0.001 | Ref  0.83  1.57  3.05  3.19 | (0.47-1.48)  (0.93-2.63)  (1.87-4.99)  (1.59-6.39) | 0.536  0.088  <0.001  0.001 |
| *Self-prioritised ethnic group*  European  Māori  Pacific  Asian  MELAA and others | 3169 (55.4)  763 (13.3)  787 (13.7)  810 (14.2)  196 (3.4) | Ref  2.50  1.65  1.03  1.22 | (2.12-2.95)  (1.40-1.94)  (0.87-1.21)  (0.91-1.64) | <0.001  <0.001  0.731  0.190 | Ref  2.48  4.23  2.45  1.39 | (1.53-4.03)  (2.87-6.21)  (1.62-3.71)  (0.55-3.54) | <0.001  <0.001  <0.001  0.484 |
| *NZDep 2006 (deciles)****  1-2 (least deprived)  3-4  5-6  7-8  9-10 (most deprived) | 947 (16.5)  1091 (19.0)  991 (17.3)  1201 (21.0)  1503 (26.2) | Ref  1.17  1.40  1.70  2.16 | (0.97-0.41)  (1.16-1.70)  (1.42-2.03)  (1.82-2.57) | 0.100  <0.001  <0.001  <0.001 | Ref  1.16  1.37  1.85  3.67 | (0.63-2.14)  (0.74-2.53)  (1.05-3.26)  (2.20-6.13) | 0.638  0.314  0.034  <0.001 |
| *Pre-pregnancy BMI (kg/m^2^)*  < 25  >25 & <30  >30 | 3029 (59.7)  1126 (22.2)  917 (18.1) | Ref  1.27  1.68 | (1.10-1.46)  (1.44-1.95) | 0.001  <0.001 | Ref  1.27  1.48 | (0.85-1.90)  (0.96-2.27) | 0.241  0.075 |
| *Physical activity before and during pregnancy*  Moderate/vigorous before and during  Moderate/vigorous only before or during  No Moderate/vigorous before and during | 1783 (33.9)  1404 (26.7)  2073 (39.4) | Ref  0.89  0.90 | (0.77-1.03)  (0.79-1.02) | 0.115  0.105 | Ref  0.75  0.82 | (0.49-1.13)  (0.57-1.18) | 0.171  0.280 |
| *Pre/during pregnancy smoking pattern*  Non-smoker  Stopped smoking  Continued smoking | 4234 (80.7)  517 (9.9)  494 (9.4) | Ref  2.01  3.49 | (1.67-2.42)  (2.85-4.26) | <0.001  <0.001 | Ref  0.97  1.18 | (0.53-1.77)  (0.61-2.28) | 0.915  0.630 |
| *Fruits and vegetables guideline adherence*  Yes  No | 1313 (25.0)  3947 (75.0) | Ref  1.11 | (0.97-1.26) | 0.115 | Ref  1.01 | (0.70-1.45) | 0.973 |
| *Breads and cereals guideline adherence*  Yes  No | 1389 (26.4)  3871 (73.6) | Ref  0.69 | (0.61-0.78) | <0.001 | Ref  0.69 | (0.49-0.97) | 0.034 |
| *Milk and derivatives guideline adherence*  Yes  No | 3061 (58.2)  2199 (41.8) | Ref  0.97 | (0.87-1.09) | 0.614 | Ref  1.19 | (0.86-1.64) | 0.285 |
| *Meats and alternatives guideline adherence*  Yes  No | 1149 (21.9)  4109 (78.1) | Ref  0.84 | (0.74-0.96) | 0.013 | Ref  0.64 | (0.45-0.91) | 0.012 |

Ref., reference category.

RRR, Relative risk ratio; NCEA, National Certificate of Education Achievement; NZDep2006**, neighborhood deprivation index 2006; *kg/m^2^*, kilograms/squared metres.

*Infants aged 7 to 12 months in the nine-month interview.

**The group of mothers whose infants had timely introduction to complementary foods was the reference group in these analyses.

*** Area-level socioeconomic deprivation was measured by using the NZ Index of Deprivation. Derived from the 2006 national census according to the methodology described in Salmond et al (34). NZDep2006 deciles 1-2 represent the least deprived neighbourhoods and deciles 9-10 the most deprived neighbourhoods.

Missing for variables listed in the table (n): sex (n=26); age in years (n=26); birth weight (n=26); breastfeeding (n=44); parity (n=40); planned pregnancy (n=62); age group (n=35); childbirth class (n=81); education (n=50); ethnicity (n=45); deprivation index (n=37); pre-pregnancy BMI (n=698); physical activity (n=510); smoking pattern (n=525); adherence to fruits and vegetables consumption (n=510); adherence to breads and cereals consumption (n=510); adherence to milk and derivatives consumption (n=510); adherence to meats and alternatives consumption (n=512).
